# Editorial: Violence and the Young: A Public Health Problem - Etiology, Epidemiology, Intervention, and Prevention

**DOI:** 10.3389/fpubh.2021.724182

**Published:** 2021-07-09

**Authors:** Lawrence T. Lam, Doug Hyun Han, Wei Zhang, Lynn Tang

**Affiliations:** ^1^Tung Wah College, Hong Kong, China; ^2^Faculty of Health, University of Technology Sydney, Sydney, NSW, Australia; ^3^Department of Psychiatry, Chung-Ang University, Seoul, South Korea; ^4^School of Medicine and Health Management, Tongji Medical College, Huazhong University of Science and Technology, Wuhan, China; ^5^Department of Sociology and Social Policy, Lingnan University, Hong Kong, China

**Keywords:** violence, prevention, epidemiology, intervention, aetiology (etiology), youth, children

Violence, particularly among young people, has long been identified as an important global public health issue by (1). With the publication of the World Report on Violence and Health (WRVH), the issue was propelled onto the international health agenda (2). To provide a platform for a better understanding of violence, facilitating data collection for epidemiological investigation, and designing intervention strategies appropriate for the prevention of violence, a framework was proposed in the WRVH (2). In this framework, there consisted of a matrix combining the broad types of violence and the nature of violence to further enhance the structural understanding of the multiplicity of the construct. Three broad types of violence reflecting the agency or the perpetrators of violence, namely self-directed, interpersonal, and collective, were included with physical, sexual, psychological, and deprivation, and neglect as the nature of violence (2). To provide further exposition on the matrix, Rutherford and colleagues also came up with a glossary for each category of violence with clear definitions (3).

In terms of the strategies for the intervention and prevention of youth violence, epidemiological data play an important role. Based on the data collected by UNICEF, it was reported that more than 170 million children under the age of five are living in a household with a mother being a victim of intimate partner violence, and over one-third of students of 13–15 years have experienced bullying. About three-quarters of children aged between 2 and 4 are subjected to violent discipline by their parents or carers regularly and about 10% of children are still not receiving proper legal protection from corporal punishment globally (4, 5).

Violence against children and young people is a complex problem, so are the solutions in tackling this global issue. Some evidence-based solutions and preventive interventions have been suggested by UNICEF (6). However, with the global COVID-19 pandemic crisis still lingering, it is anticipated that the situation will be greatly affected by a multitude of potential causes such as the confinement to a limited space due to the lockdown containment measure, unemployment due to a low level of economic activities, shortage of resources in supporting the necessary services in providing protection and relief to children, young people and parents (7). As such, greater efforts in research are required in searching for intervention strategies in the current global environment.

This Research Topic aims to draw together a group of researchers with a keen interest in youth violence prevention and intervention to explore the topic from diverse disciplinary and methodological backgrounds.

In this series of articles, Shin et al. studied the symptomatology of the adolescent Complex- Post Traumatic Stress Disorders (C-PTSC). It was found that young people who suffered from C-PTSD were more likely to have a history of sexual assault, dissociation, and self-harm in comparison to those who had less complex PTSD. While still on the topic of PTSD, another group of researchers recognized that PTSD was a common psychopathological sequela in young people who had been physically or sexually abused in Germany. Utilizing data collected from a randomized controlled trial, Dams et al. found that the consequence of the abuse had affected the Health-related Quality of Life of young people, causing significant delay and productivity losses in education, and exerting a large economic burden on society.

In terms of the environmental risk factor of physical and sexual abuse among children and adolescents, the family environment has been identified as high risk. There has been a good volume of studies conducted on the topic of family violence, particularly in the developed countries (8). However, notwithstanding the preventive measures put in place by the international bodies, such as the UN and WHO, family violence is still a significant problem of child health in many underdeveloped countries. As such, Addae and Tang and her student undertook a study on family violence in Ghana. Of particular interest is the lived experience of young people being exposed to violence in their own homes against themselves or close family members. Results of the study indicated that family violence has been legitimized by some socio-ecological factors, such as patriarchy, the normalization of corporal punishment as a method of child discipline, and superstitious beliefs about health.

The rapid development of Information and Communication Technologies, particularly the Internet, has provided great benefits to humankind, but also exposed young people to the virtual environment that could easily be abused with malicious intent. Cyberbullying among young people has long been recognized as a problem to adolescent health (9). An ample of studies have been conducted on the topic among older adolescents since they tend to be of a more at-risk population. However, a growing trend in cyberbullying among children and young adolescents has also been observed in recent years (10). Zhu et al. have conducted a comprehensive review of the recent literature in examining the prevalence and risk factors, as well as exploring preventive measures of cyberbullying in young people.

Suicide is the most severe form of intentional self-harm and youth suicide is one of the important topics in adolescent health. In terms of the intervention opportunity, the emergency department is one of the clinical venues where effective intervention could be applied when young people receive medical treatments for intentional self-harm or self-injury. Kim et al. developed the multi-disciplinary emergency consultation system (MECS) for suicide attempters with drug overdose as an intervention program implemented at the hospitals. His study also demonstrated that a rapid response of a multidisciplinary team for young suicide attempters could reduce unnecessary ICU treatment and overall medical costs.

We hope this series of articles can draw attention to violence toward children and young people and call for further actions from governments all over the world to take effective measures to end current violence in all forms and to prevent further violence against the vulnerable population under their care. As the world is ever-changing and so does violence against young people, thus ongoing efforts are required to keep researching for appropriate preventive measures and to provide the best evidence for policymaking across different cultural, economic, and political milieu.

## Author Contributions

All authors listed have made a substantial, direct and intellectual contribution to the work, and approved it for publication.

## Conflict of Interest

The authors declare that the research was conducted in the absence of any commercial or financial relationships that could be construed as a potential conflict of interest.
